# Changes in Secure Messaging After Implementation of Billing E-Visits by Demographic Group

**DOI:** 10.1001/jamanetworkopen.2024.27053

**Published:** 2024-08-09

**Authors:** A. Jay Holmgren, Carrie K. Grouse, Aris Oates, Julie O’Brien, Maria E. Byron

**Affiliations:** 1 Division of Clinical Informatics and Digital Transformation, University of California, San Francisco; 2 Department of Neurology, University of California, San Francisco; 3 Department of Pediatrics, University of California, San Francisco; 4 Department of Medicine, University of California, San Francisco

## Abstract

This cohort study investigates the association of demographic characteristics with changes in patient portal messaging after implementation of e-visit billing.

## Introduction

Beginning in 2020, the Centers for Medicare & Medicaid Services and private payers expanded billing options for asynchronous patient portal messages, known as e-visits. Billing requirements include medical decision-making and a minimum of 5 minutes of clinician time over 7 days. Several large health systems recently implemented billing messages as e-visits,^1^ possibly in response to increased message volume after the COVID-19 pandemic began in March 2020.^2^ Early research showed that the potential to receive a bill was associated with a small decrease in overall message volume.^3^ However, little is known regarding demographic characteristics of patients with reduced messaging. It is critical to understand whether the association of e-visit billing with patient messaging differed by race and ethnicity, language, age, or payer.^4^ We assessed changes in patient-initiated messaging volume after e-visit billing implementation across patient demographics at a large health system in a primarily fee-for-service environment.

## Methods

This cohort study was deemed exempt from review and consent by the University of California, San Francisco (UCSF) Institutional Review Board for using deidentified, aggregate data and follows STROBE reporting guidelines. We analyzed e-visit billing at UCSF Health, which began clinician-driven e-visit billing on November 14, 2021, with patients informed via the portal before sending a message that it may result in a bill and provided information about cost sharing.

To capture the association of e-visit implementation with patient messaging rates across demographic groups, we measured total patient medical advice request messages weekly from October 1, 2020, to August 20, 2022, with November 14, 2021, as the implementation date. The sample period ended on August 20, 2022, to avoid confounding given that UCSF implemented an unrelated change in the portal interface the following week. We measured total weekly messages by patient race and ethnicity (as indicated in the electronic health record), preferred language, payer category (commercial, California state Medicaid [Medi-Cal], Medicare, or other [primarily self-pay]), and age. Messages include those sent by patients or designated proxies. We used 2-tailed *t* tests with unequal variances to assess differences in mean weekly messaging before and after e-visit implementation. All analyses were conducted in Stata version 17.0 (StataCorp).

## Results

Among 5 558 460 messages, mean weekly messages decreased by 2.0% over 94 weeks. Across race and ethnicity groups, the largest reductions in mean weekly messages after e-visit implementation were among Latinx (−323.4 messages; 95% CI, −584.6 to −62.3 messages [−5.3%]; *P* = .02) and Asian (−506.5 messages; 95% CI, −940.5 to −72.5 [−5.1%]; *P* = .02) patients (Table).

**Table.** Weekly Messaging Volume

Demographic group	Messages			Change, %	*P* value^c^
	Weekly volume, mean (SD)		Difference (95% CI)		
	Before implementation^a^	After implementation^b^			
Total	59 648 (5487)	58 436 (5695)	−1212.1	−2.0	.30
Race and ethnicity^d^					
Asian	9900 (1140)	9393 (972)	−506.5 (−940.5 to −72.5)	−5.1	.02
Black	2464 (255)	2437 (241)	−26.2 (−128.7 to 76.4)	−1.1	.61
Latinx	6125 (598)	5802 (651)	−323.4 (−584.6 to −62.3)	−5.3	.02
White	35028 (3151)	34658 (3411)	−369.5 (−1740.7 to 1001.8)	−1.1	.59
Other	6132 (638)	6146 (613)	13.5 (−245.2 to 272.1)	0.2	.92
Preferred language					
Non-English language	1611 (198)	1568 (180)	−42.3 (−120.3 to 35.6)	−2.6	.28
English	51 906 (4753)	50 722 (4997)	−1183.2 (−3214.6 to 848.1)	−2.3	.25
Payer					
Commercial	31 702 (3009)	30 741 (3208)	−960.6 (−2257.7 to 336.5)	−3.0	.14
Medicare	19 642 (2005)	19 692 (1848)	49.3 (−745.3 to 844.0)	0.3	.90
Medi-Cal	6526 (682)	6482 (659)	−44.4 (−321.9 to 233.1)	−0.7	.75
Other (eg, self-pay)	1778 (722)	1522 (144)	−256.5 (−458.4 to −54.7)	−14.4	.01
Age, y					
0-11	4006 (598)	4218 (678)	211.7 (−56.2 to 479.6)	5.3	.12
12-17	2285 (392)	2303 (351)	18.1 (−134.9 to 171.1)	0.8	.81
18-49	21 093 (2572)	18 921 (1872)	−2172.4 (−3083.0 to −1261.9)	−10.3	<.001
50-65	15 754 (1398)	15 421 (1447)	−333.6 (−925.5 to 258.3)	−2.1	.27
66-80	14 032 (1580)	14 907 (1427)	875.2 (255.8 to 1494.6)	6.2	.01
≥80	2478 (325)	2667 (298)	188.9 (60.7 to 317.1)	7.6	<.001

Abbreviation: Medi-Cal, California state Medicaid.

^a^ November 1, 2020, to November 13, 2021.

^b^ November 14, 2021, to August 20, 2022.

^c^ Statistical significance was set at α = 0.05.

^d^ Race and ethnicity data are defined from electronic health record data. The other category aggregates patients who are designated as American Indian or Alaska Native, Native Hawaiian or Other Pacific Islander, Southwest Asian and North African, multiracial or multiethnic, other (unspecified), and unknown or declined.

Across payers, patients in the other category saw a reduction of −256.5 messages (95% CI, −458.4 to −54.7 messages [14.4% ]; *P* = .01). Patients ages 18 to 49 years saw the largest reduction across age groups (−2172.4 messages; 95% CI, −3083.0 to −1261.9 messages [−10.3%]; *P* < .01), while patients aged 66 to 80 and older than 80 years had increased messages. Trends were stable throughout the postimplementation period (Figure).

**Figure.**
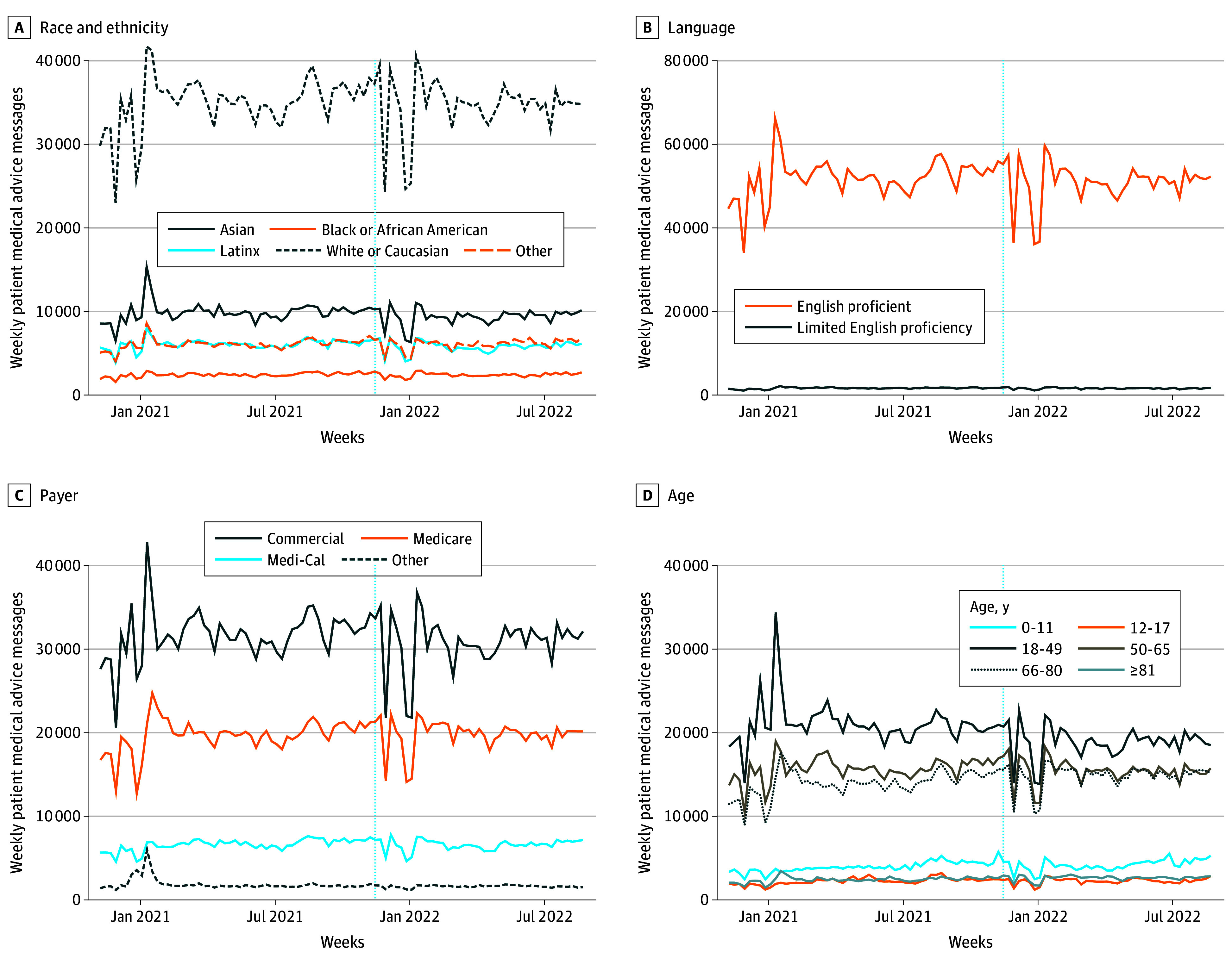
Weekly Medical Advice Request Messaging Volume Vertical dashed lines represent November 2021 e-visit billing implementation. Medi-Cal indicates California state Medicaid.

## Discussion

This cohort study found several differences in the change in messaging across patient demographic characteristics. Patients with self-pay and those aged 18 to 49 years saw the largest decreases in mean weekly message volume, and Latinx and Asian patients saw a slightly larger decrease than other racial and ethnic groups. Some patients, including those older than 65 years, had increased messaging volume. While it is reassuring that we did not find evidence of differential associations with messaging for Medicaid or older patients, groups with lower rates of prepandemic messaging,^5,6^ the larger reduction in messages for some patient groups warrants further investigation.

Limitations include data from a single health system and an inability to assess care delivered outside of UCSF, associations with patient outcomes, or causality. Our aggregate data allowed us to assess only high-level changes in messaging across groups, which may reflect changes in patient behavior after they learn of the possibility of billing for messaging. We could not observe whether a patient received an e-visit bill or faced out-of-pocket costs. Future research should investigate the association of billing for technology-enabled care with health across patient demographics.

## Notes

Supplement.
Data Sharing Statement
