# Supplementary material for: Changes in Secure Messaging After Implementation of Billing E-Visits by Demographic Group
Source: JAMA Netw Open. 2024 Aug 9;7(8):e2427053. doi: 10.1001/jamanetworkopen.2024.27053 (PMC11316232; doi:10.1001/jamanetworkopen.2024.27053)
Supplement: Supplement. — Data Sharing Statement [file jamanetwopen-e2427053-s001.pdf]

Data Sharing Statement

Holmgren. Changes in Secure Messaging After Implementation of Billing E-Visits by Demographic Group. JAMA Netw Open. Published online August 9, 2024. doi:10.1001/jamanetworkopen.2024.27053

Data

Data available: No

Additional Information

Explanation for why data not available: Patient-level data is protected health information and cannot be shared per institutional data rules.
